# ZnFeAl-layered double hydroxides/TiO_2_ composites as photoanodes for photocathodic protection of 304 stainless steel

**DOI:** 10.1038/s41598-018-22572-7

**Published:** 2018-03-07

**Authors:** Xiu-tong Wang, Xiao-bo Ning, Qian Shao, Sheng-song Ge, Zhi-ying Fei, Jing Lei, Bao-rong Hou

**Affiliations:** 1 Key Laboratory of Marine Environmental Corrosion and Bio-fouling, Institute of Oceanology, Chinese Academy of Sciences, Qingdao, 266071 China; 2 College of Chemical and Environmental Engineering, Shandong University of Science and Technology, Qingdao, 266590 China; 3 Open Studio for Marine Corrosion and Protection, Qingdao National Laboratory for Marine Science and Technology, Qingdao, 266237 China

## Abstract

A series of ZnFeAl-layered double hydroxides/TiO_2_ (ZnFeAl-LDHs/TiO_2_) composites are synthesized by a combined anodization and hydrothermal method. The structure, surface morphology, photo absorption and photocathodic protection properties of these samples are characterized by X-ray diffraction (XRD), scanning electron microscopy (SEM), X-ray photoelectron spectroscopy (XPS), ultraviolet-visible diffuse reflectance spectroscopy (UV-vis DRS) and electrochemical tests. The unique structure of the ZnFeAl-LDHs reduces the charge carriers recombination, and the visible photoresponse property increase the light harvesting. The XPS study reveals that the electrons in the ZnFeAl-LDHs travel to TiO_2_, and the ZnFeAl-LDHs/TiO_2_ composites generate and transfer more electrons to 304 stainless steel (304SS), and exhibits a better photocathodic protection performance than pure TiO_2_. In addition, after intermittent visible-light illumination for four days, the photoanode still exhibits good stability and durability.

## Introduction

Stainless steels are used in many fields because of their good corrosion resistance. However, due to the effects of chloride ions and marine microorganisms, pitting corrosion easily occurs and the passivation film of stainless steel tends to be destroyed in marine environments, accelerating corrosion^1,2^. Several methods have been proposed to slow down the corrosion rate of stainless steels^3,4^, and photocathodic protection is one of the most innovative methods.

TiO_2_ is a low cost, non-toxic, highly stable semiconductor. Since the first report of photoelectrochemical water splitting using a TiO_2_ electrode under ultraviolet light^5^, the photoelectric effect of TiO_2_ has attracted the extensive attention and research^6–10^. In recent years, the photocathodic protection effect of TiO_2_ has attracted the interests of scientists. Y. Ohko^11^ and T. Imokawa^12^ investigated the photoelectrochemical behavior of 304 stainless steels coated with TiO_2_, and Yuan *et al*.^13^ investigated the photocathodic protection of Cu using a TiO_2_ coating. Previous researches have indicated that TiO_2_ is excited and generates electron-hole pairs under light illumination, and the electrons can be transferred to metals via the conduction band. This allows the potential of metals to be more negative than the corrosion potential, inhibiting the corrosion of the metals^14^. However, the sunlight utilization rate and photon separation rate of TiO_2_ are low^15^, which limits the practical applications of TiO_2_. Efforts have been devoted to modifying the band structure of TiO_2_ by cation doping or semiconductor doping^16–20^. Semiconductor doping is an effective method to promote photoelectrochemical properties, and the most reported semiconductor materials include WO_3_, CdS and SnO_2_^21,22^. However, some layered double hydroxides compounds (or hydrotalcite-type compounds) have been recently shown to have similar effects on semiconductors.

Layered double hydroxides (LDHs, [M^2+^_1-x_M^3+^_x_(OH)_2_]^x+^[(A^n−^)_x/n_]^x−^·mH_2_O), which contain cationic metal layers and charge-balancing anions in the inter-layer regions, have attracted considerable interest. They are widely used as catalysts^23–25^, anion exchangers^26^, energy storage materials^27,28^ and adsorbents^29^, but they have not been applied in photoelectric anticorrosion of stainless steels. LDHs based on zinc oxides have shown strong visible light absorption^30,31^. The oxo-bridges in Fe-based LDH photocatalysts help to inhibit the recombination of electrons with holes, and extend the diffusion length of the hole^32–34^. Additionally, Mantilla discovered that ZnAlFe-LDH materials show semiconductor properties in the UV-vis region after heat treatment^35^. In this work, we synthesized ZnFeAl-LDHs/TiO_2_ photoanodes and investigated their photocathodic protection of 304 stainless steels.

## Methods

### Synthesis of TiO_2_ nanotubes

TiO_2_ nanotubes were synthesized by electrochemical anodization method. First, titanium foils (BaoTi Group Co., Ltd.) with a size of 40 mm × 10 mm × 0.3 mm were polished in a mixture of NH_4_F (3 wt.%, Sinopharm Chemical Reagent Co., Ltd.), H_2_O (17.2 vol.%, 18.2 MΩ·cm), H_2_O_2_ (41.4 vol.%, Sinopharm Chemical Reagent Co., Ltd.), and HNO_3_ (41.4 vol.%, Yantai SanHe Chemical Reagent Co., Ltd.) for 30 s after an ultrasonic cleaning in ethanol and distilled water for 10 min, and then they were rinsed with ethanol and deionized water several times. The anodization process was carried out in an electrolyte system containing 0.44 g of NH_4_F (Sinopharm Chemical Reagent Co., Ltd.), 8 mL of deionized water, and 80 mL of glycol (Sinopharm Chemical Reagent Co., Ltd.) at 20 V for 1.5 h, using a Pt plate (20 mm × 20 mm × 0.3 mm) as the counter electrode and titanium foil as the working electrode. Finally, the samples were annealed at 450 °C for 2 h in air at a heating rate of 5 °C/min. All chemicals were analytical reagent grade and used without further purification.

### Preparation of ZnFeAl-LDHs/TiO_2_ composites

The ZnFeAl-LDHs/TiO_2_ composites were prepared by hydrothermal method. First, 3.0 mmol of Zn(NO_3_)_2_·6H_2_O (Sinopharm Chemical Reagent Co., Ltd.), 0.1 mmol of Fe(NO_3_)_3_·9H_2_O (Sinopharm Chemical Reagent Co., Ltd.), 0.9 mmol of Al(NO_3_)_3_·9H_2_O (Sinopharm Chemical Reagent Co., Ltd.) and 14 mmol of urea (Sinopharm Chemical Reagent Co., Ltd.) were dissolved into 50 ml of deionized water solution, and stirred for 10 min at room temperature. Then, the pH value was adjusted to 3.5 with a NaOH (0.6 M, Sinopharm Chemical Reagent Co., Ltd.) solution and stirred for another 10 min. The solution was transferred to a Teflon-lined autoclave. Finally, the prepared TiO_2_ nanotubes were placed in the autoclave at 120 °C for 8 h. The samples were taken out and washed several times with deionized water and ethanol. The above description is the synthetic process of ZnFeAl-LDHs/TiO_2_ with total metal concentration of 80 mmol/L. In this experiment, three samples were synthesized with a total metal concentration of 40 mmol/L, 80 mmol/L and 160 mmol/L, and the samples were designated ZnFeAl-LDHs/TiO_2_(a), ZnFeAl-LDHs/TiO_2_(b), and ZnFeAl-LDHs/ TiO_2_(c), respectively.

### Characterization

The XRD patterns were recorded on D/Max 2550 diffractometer with Cu *Kα* radiation in the 2*θ* range from 10° to 70°. The SEM images were obtained by a HITACHI S-4800 scanning electron microscope. The EDS spectrum were obtained by an Oxford INCA Energy 350 energy dispersive X-ray spectrometer. The UV-vis absorption spectra were recorded with a HITACHI U-4100 spectrophotometer. The XPS data were recorded on a Perkin-Elmer PHI-1600 ESCA spectrometer employing Mg *Kα* X-rays.

### Electrochemical Measurements

**Figure 1:**
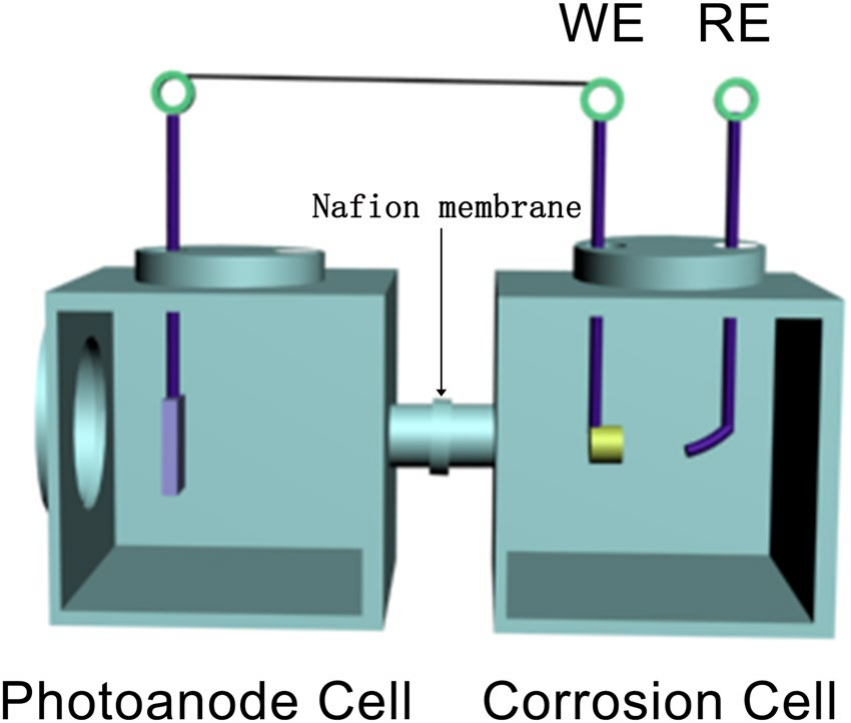
A coupling system with two cells for the electrochemical measurements. The cell on the left is Photoanode Cell, the electrode in the cell was ZnFeAl-LDHs/TiO_2_. The cell on the right is Corrosion Cell, the electrodes in the cell were 304SS and a saturated calomel electrode. The electrodes, ZnFeAl-LDHs/TiO_2_ and 304SS were connected by a wire as the working electrode (WE). The saturated calomel electrode was used as the reference electrode (RE).

An electrochemistry working station (PARSTAT 4000+, Princeton, USA) was used for the electrochemical test of the open circuit potential (OCP) and photocurrent density. The measurement of the OCP was evaluated in a two-cell system includes corrosion cell and photoanode cell. The corrosion cell and photoanode cell are connected, and there is a Nafion membrane at the joint of two cells (Fig. 1). The electrodes in the corrosion cell were 304SS and a reference electrode (RE, saturated calomel electrode), and the solution was 3.5 wt.% NaCl, the electrode in the photoanode cell was ZnFeAl-LDHs/TiO_2_, and the solution was a mixture of 0.1 mol/L Na_2_S (Shanghai TongYa Chemical Technology Development Co., Ltd.) and 0.1 mol/L Na_2_SO_3_ (Sinopharm Chemical Reagent Co., Ltd.). In addition, the electrodes, ZnFeAl-LDHs/TiO_2_ and 304SS were connected by a wire as the working electrode (WE). The measurement of the photocurrent density was evaluated in a single-cell system with traditional three electrodes (Pt as the counter electrode), and the solution was a mixture of 0.1 mol/L Na_2_S and 0.1 mol/L Na_2_SO_3_. A Xenon lamp (PLS-SXE 300 C, Beijing Perfectlight Company, China) with a 400 nm glass filter was used as the light source device.

### Data Availability

All data generated or analysed during this study are included in this published article.

## Results and Discussion

**Figure 2:**
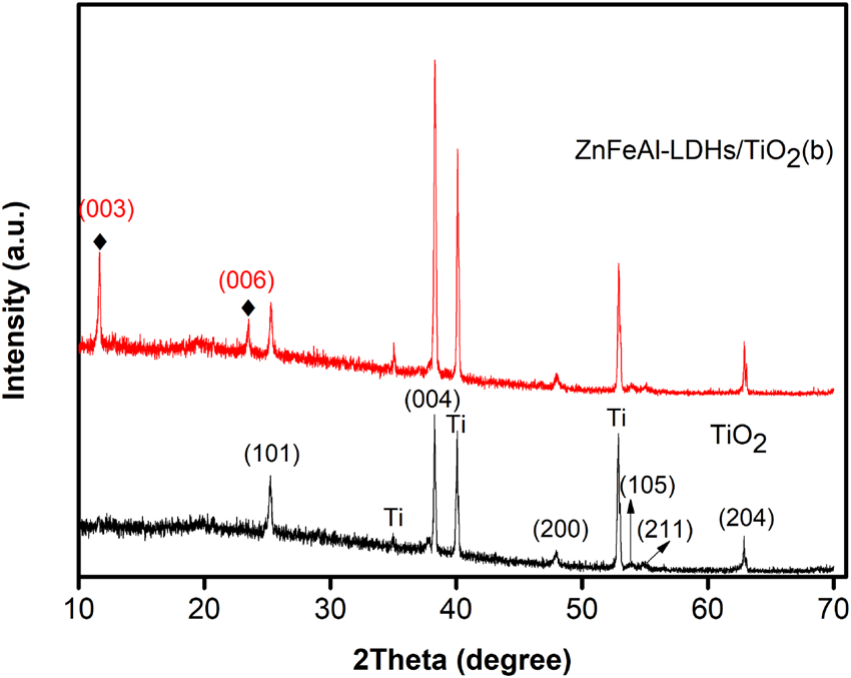
XRD patterns of the TiO_2_ and ZnFeAl-LDHs/TiO_2_(b). The black line represents the XRD pattern of TiO_2_, the red line represents the XRD pattern of ZnFeAl-LDHs/TiO_2_(b).

The XRD patterns of TiO_2_ and the ZnFeAl-LDHs/TiO_2_ composites are shown in Fig. 2. The characteristic diffraction peaks at 2*θ* = 25.3°, 37.8°, 48.0°, 53.9°, 55.1° and 62.7° belong to anatase phase TiO_2_ (JCPDS 21-1272). The characteristic diffraction peaks at 2*θ* = 35.1°, 40.2°, and 53.0° belong to pure Ti. The characteristic diffraction peaks at 2*θ* = 11.6° and 23.3° belong to ZnFeAl-LDHs/TiO_2_(b), and correspond to the characteristic (003) and (006) reflections of a hydrotalcite phase. The (003) reflection is a typical peak of hydrotalcite-type materials. This proves the formation of layered double hydroxides in the hydrotalcite structure.

**Figure 3:**
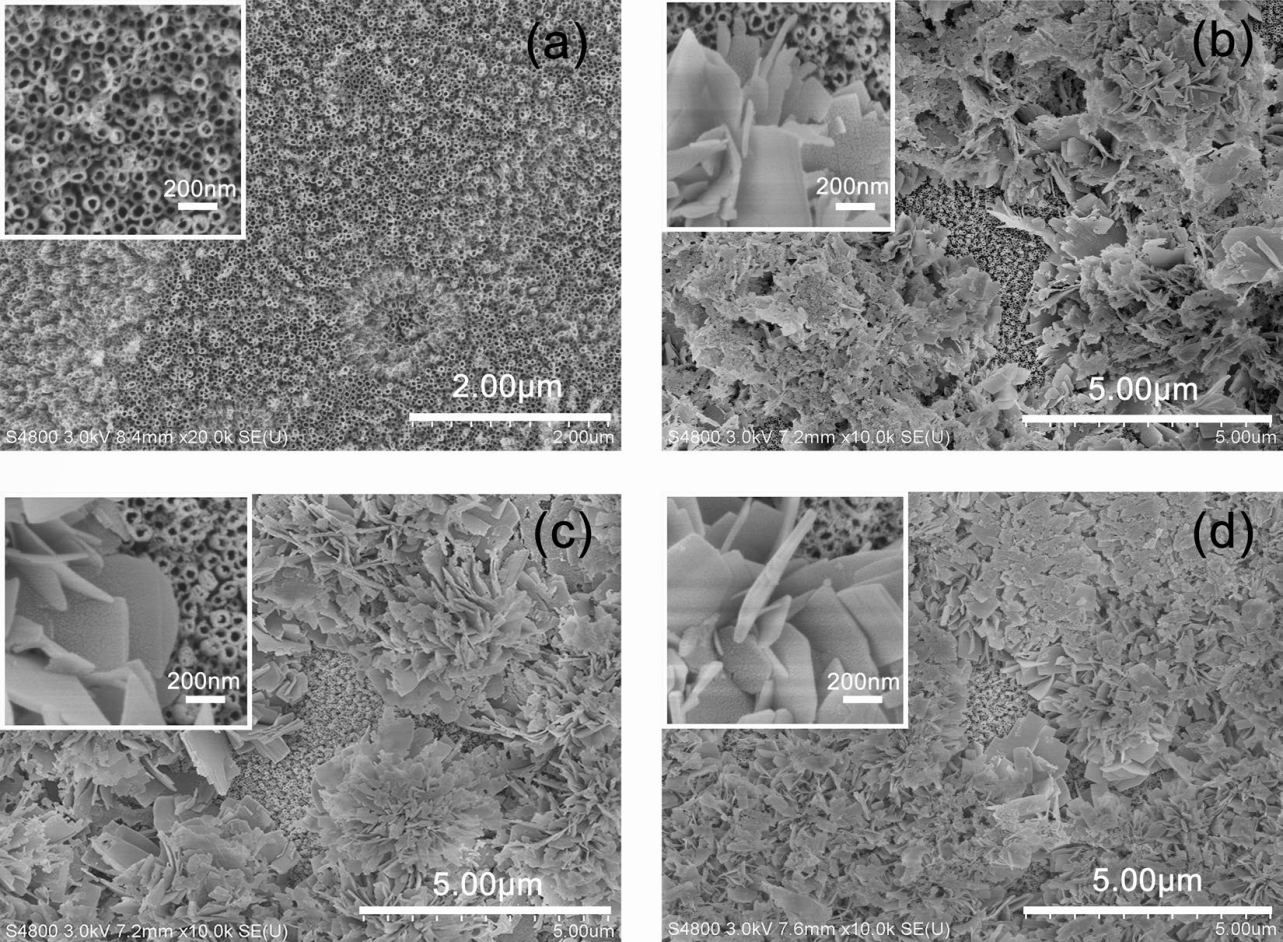
(**a**) SEM image of TiO_2_, (**b**) SEM image of ZnFeAl-LDHs/TiO_2_(a), (**c**) SEM image of ZnFeAl-LDHs/TiO_2_(b) and (**d**) SEM image of ZnFeAl-LDHs/TiO_2_(c). The figures in the upper left corner are magnified views.

**Figure 4:**
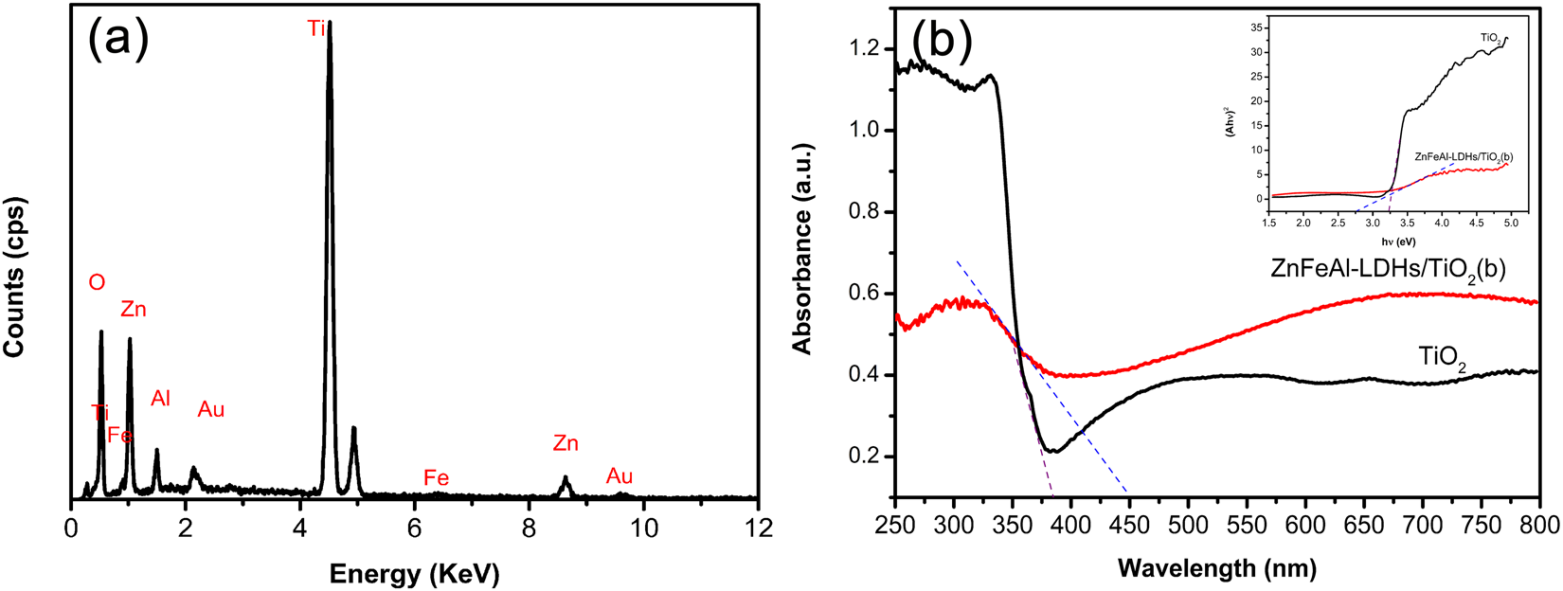
(**a**) EDS spectrum of ZnFeAl-LDHs/TiO_2_(b), and (**b**) UV-vis DRS of TiO_2_ and ZnFeAl-LDHs/TiO_2_(b). In Fig. 4. (**b**), The black line represents the UV-vis DRS of TiO_2_, the red line represents the UV-vis DRS of ZnFeAl-LDHs/TiO_2_(b), the purple and blue dotted lines are tangent lines. The figure in the upper right corner of Fig. 4. (**b**) represents the band gaps of TiO_2_ and ZnFeAl-LDHs/TiO_2_(b).

The SEM images of TiO_2_ and the ZnFeAl-LDHs/TiO_2_ composites are shown in Fig. 3. The TiO_2_ nanotubes have an orderly array structure, and the diameter of the nanotube is approximately 70 nm. In the ZnFeAl-LDHs composites, the ZnFeAl-LDHs material is supported on the TiO_2_ nanotubes in a lamellar form with the length of 400–800 nm. In ZnFeAl-LDHs/TiO_2_(a), some ZnFeAl-LDHs did not form a lamellar structure and aggregation occurred (Fig. 3(b)), the aggregation of ZnFeAl-LDHs will inevitably affect the light absorption and the transportation of photoelectrons. In ZnFeAl-LDHs/TiO_2_(b) and ZnFeAl-LDHs/TiO_2_(c), ZnFeAl-LDHs nanoflakes are observed to be cross-distributed on the surface of TiO_2_, and there are more nanoflakes on ZnFeAl-LDHs/TiO_2_(c) (Fig. 3(d)) than ZnFeAl-LDHs/TiO_2_(b) (Fig. 3(c)), in Fig. 3(c), the ZnFeAl-LDHs nanoflakes on the surface of TiO_2_ nanotubes distribute more closely, which may influence the light absorption and electron generation of TiO_2_, then influence the photocathodic protection performance. In addition, the EDS spectrum of ZnFeAl-LDHs/TiO_2_(b) is shown in Fig. 4(a). It can be seen that the sample consisted of Zn, Fe, Al, Ti and O, and the chemical composition agreed well with that of ZnFeAl-LDHs/TiO_2_.

The UV-vis spectra of TiO_2_ and ZnFeAl-LDHs/TiO_2_(b) are shown in Fig. 4(b). It can be seen that pure TiO_2_ exhibits a steep absorption edge at approximately 380 nm. In addition, a red shift of the absorption edge is observed in ZnFeAl-LDHs/TiO_2_(b), inducing stronger light absorption in the visible region. And the band gap of the two samples were achieved followed the equation,

1 $${(\mathrm{Ah}{\rm{\nu }})}^{{\rm{2}}}={\rm{K}}{(h{\rm{\nu }}-{\rm{Eg}})}^{n/2}$$

where A, h, υ, K, and Eg are the absorption coefficient, planck constant, light frequency, proportionality constant, band gap, respectively. The values of the band gap are obtained by extending the vertical segment to hυ axis. We can see that the band gaps of TiO_2_ and ZnFeAl-LDHs/TiO_2_(b) are approximately 3.2 and 2.8 eV, respectively. Compared to pure TiO_2_, the ZnFeAl-LDHs/TiO_2_ composite exhibits a narrower band gap, which can enhance the photo absorption.

**Figure 5:**
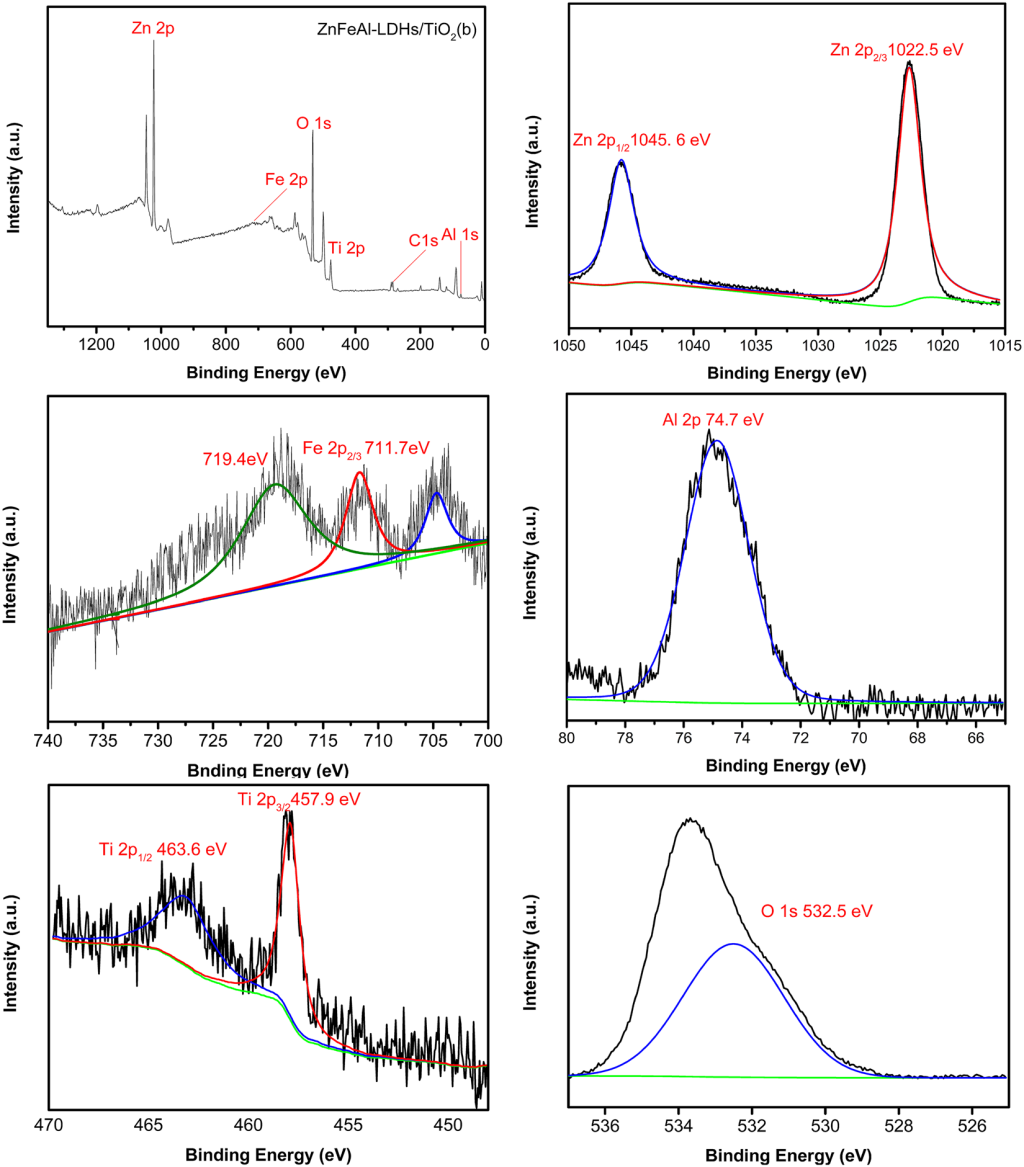
XPS spectra of ZnFeAl-LDHs/TiO_2_(b). Figure 5 represents the total spectrum of ZnFeAl-LDHs/TiO_2_(b) and the spectra of the elements Zn, Fe, Al, Ti, O.

The XPS spectra of ZnFeAl-LDHs/TiO_2_(b) are shown in Fig. 5. All the elements Zn, Fe, Al, Ti and O are detected, and the observed peaks at 1045.6 eV and 1022.5 eV are localized in the Zn 2p_1/2_ and 2p_3/2_ regions. We can see that the sample shows the main Fe 2p_3/2_ peak at 711.7 eV, which is accompanied by a shake-up satellite line at 719.4 eV, indicating that the iron in ZnFeAl-LDHs is mainly in the form of Fe^3+^^36,37^. The observed peak at 74.7 eV of Al 2p confirmed the presence of Al^3+^, and the observed peaks at 463.6 eV and 457.9 eV correspond to Ti 2p_1/2_ and Ti 2p_3/2_, and are assigned to Ti^4+^ in TiO_2_, indicating that the main chemical state of Ti is +4. Both peaks shift to lower binding energy levels compared to those of standard peaks (464.6 eV, 458.7 eV) of TiO_2_, indicating electron transfer from ZnFeAl-LDHs to TiO_2_^38^. The observed peak at 532.5 eV of O 1 s corresponds to the oxygen species in the hydroxide form in the LDH structure^39^.

**Figure 6:**
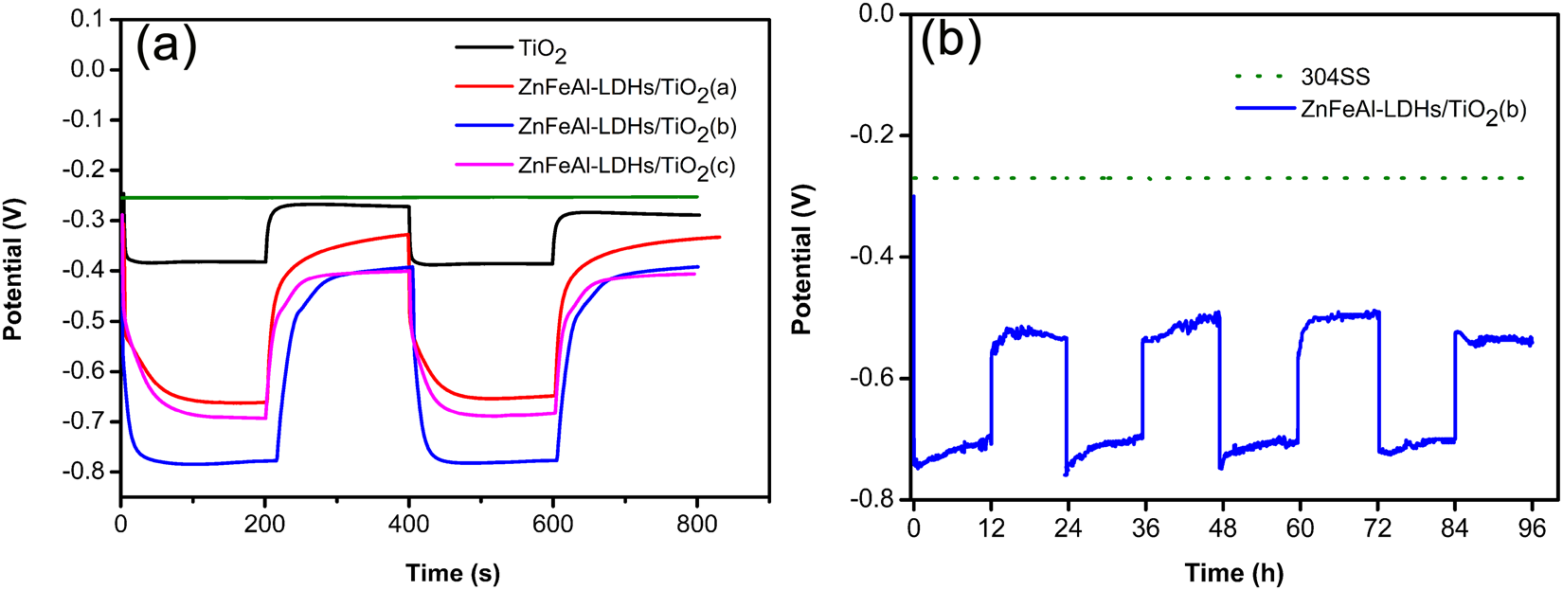
OCP-time curves of 304SS, 304SS coupled with TiO_2_ and ZnFeAl-LDHs/TiO_2_ composites. The green line represents the curve of Ecorr, and Ecorr represents the corrosion potential of 304SS. The black line, red line, blue line and pink line represent OCP-time curves of 304SS coupled with TiO_2_, ZnFeAl-LDHs/TiO_2_(a), ZnFeAl-LDHs/TiO_2_(b) and ZnFeAl-LDHs/TiO_2_(c), respectively.

**Figure 7:**
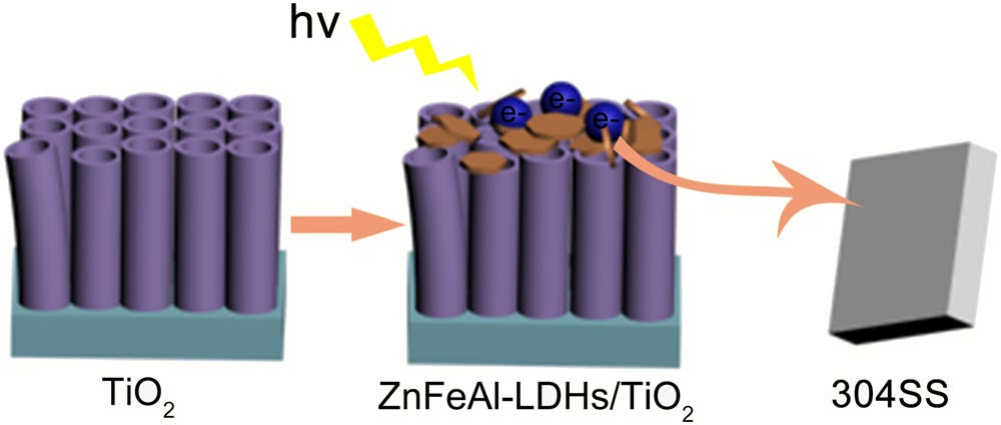
A schematic illustration for the fabrication of ZnFeAl-LDHs/TiO_2_ composites for photocathodic protection of 304SS. “hν” represent enegy, the arrows represent the transfer direction of electrons.

Figure 6(a) shows the OCP-time curves of 304SS coupled to TiO_2_ and the ZnFeAl-LDHs/TiO_2_ composites under intermittent visible-light illumination. The results show that the corrosion potential of 304SS (Ecorr) is approximately −270 mV, when 304SS is coupled to TiO_2_, the potential decrease to −380 mV when the light is switched on, and the potential increase and is close to the corrosion potential of bare 304SS when the light is off. When 304SS is coupled to the ZnFeAl-LDHs/TiO_2_ composites, the potential exhibits more obvious decrease when the light is switched on, and more negative than that of the bare 304SS when the light is off. These results indicate that when 304SS is coupled to the ZnFeAl-LDHs/TiO_2_ composites, the visible-light absorption characteristics of the ZnFeAl-LDHs/TiO_2_ composites allow the composites to absorb more visible light and generate more carriers. Additionally, the oxo-bridges in ZnFeAl-LDHs/TiO_2_ help prevent the recombination of holes with electrons, and the synergistic effect leads to more protection electrons being transferred to the 304SS. (The schematic illustration is shown in Fig. 7.) ZnFeAl-LDHs material was supported on the TiO_2_ nanotubes by hydrothermal method. Under light irradiation, both TiO_2_ and ZnFeAl-LDHs can be excited to generate electrons and holes, the electrons of the ZnFeAl-LDHs can transferred to the TiO_2_, and then transferred to 304SS to provide an protection. However, the three samples exhibit different potential changes caused by the amount and morphology of ZnFeAl-LDHs. To verify the stability and durability of the samples, the OCP-time curves of 304SS coupled with ZnFeAl-LDHs/TiO_2_(b) are investigated for 4 days with 4 cycles under intermittent visible light irradiation. Each cycle includes 12 h of light-on and 12 h of light-off. Figure 6(b) shows that the potential decrease to approximately −750 mV under illumination and remain below –700 mV after 12 h. When the light is off, the potential rises to about −500 mV, and is more negative than the corrosion potential of 304SS, which shows that the sample can provide protection in the dark. After 4 days, the sample retains a good protection performance. The results indicate that the ZnFeAl-LDHs/TiO_2_(b) photoanode exhibits good stability and durability and can provide long-term protection for 304SS.

**Figure 8:**
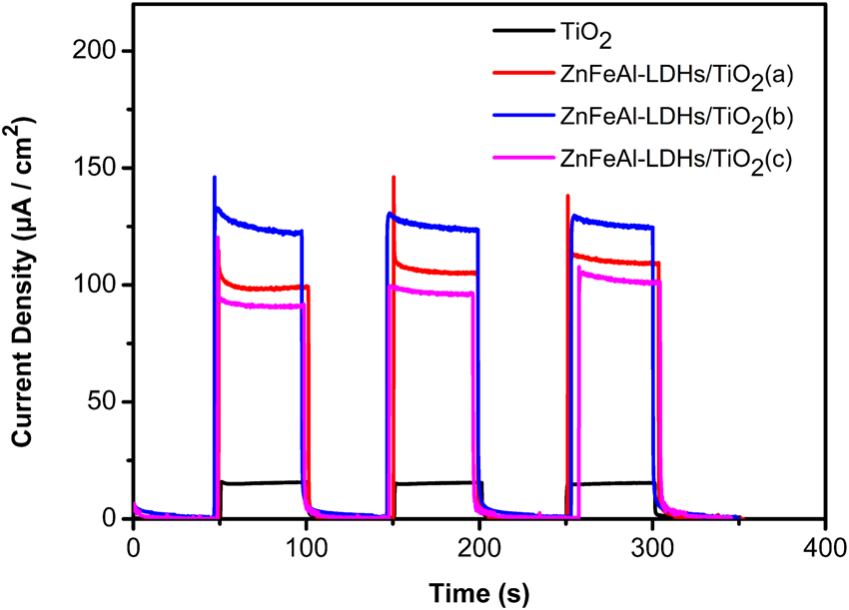
Photocurrent density curves of TiO_2_ and ZnFeAl-LDHs/TiO_2_ composites. The black line, red line, blue line and pink line represent the photocurrent density curves of TiO_2_, ZnFeAl-LDHs/TiO_2_(a), ZnFeAl-LDHs/TiO_2_(b) and ZnFeAl-LDHs/TiO_2_(c), respectively.

Figure 8 shows the photocurrent density curves of TiO_2_ and the ZnFeAl-LDHs/TiO_2_ composites under intermittent visible-light illumination. The maximum photocurrent density of ZnFeAl-LDHs reaches 138 μA/cm^2^, and the values of all the ZnFeAl-LDHs/TiO_2_ composites are larger than that of TiO_2_, which indicate that the ZnFeAl-LDHs/TiO_2_ composites generate more electrons.

## Conclusions

In summary, ZnFeAl-LDHs/TiO_2_ composites with various concentrations of ZnFeAl-LDHs are synthesized. All the composites exhibit better photocathodic protection performances for 304SS than pure TiO_2_ which is attributed to the synergistic mechanism of their unique structure and visible-light response property. The composite with a concentration of 80 mmol/L of ZnFeAl-LDHs exhibits the best performance, and the protection potential reaches –760 mV under visible-light illumination, and is lower than the corrosion potential of 304SS in the dark. Moreover, the composites have good stability and durability, this work provides a probable approach for effective and stable photocathodic protection of marine metal.
